# Do you see what I see? Optical morphology and visual capability of ‘disco’ clams (*Ctenoides ales*)

**DOI:** 10.1242/bio.024570

**Published:** 2017-04-10

**Authors:** Lindsey F. Dougherty, Richard R. Dubielzig, Charles S. Schobert, Leandro B. Teixeira, Jingchun Li

**Affiliations:** 1 Department of Integrative Biology, University of California Berkeley, 3040 VLSB #3140, Berkeley, CA 94720, USA; 2 Department of Ecology and Evolutionary Biology, University of Colorado Boulder, 334 UCB, Boulder, CO 80309, USA; 3 Museum of Natural History, University of Colorado Boulder, 265 UCB, Boulder, CO 80302, USA; 4 Pathobiological Sciences Department School of Veterinary Medicine, University of Wisconsin, Madison, 2015 Linden Drive, Madison, WI 53706, USA

**Keywords:** Flashing, Eyes, Vision, Bivalve, Signal

## Abstract

The ‘disco’ clam *Ctenoides ales* (Finlay, 1927) is a marine bivalve that has a unique, vivid flashing display that is a result of light scattering by silica nanospheres and rapid mantle movement. The eyes of *C. ales* were examined to determine their visual capabilities and whether the clams can see the flashing of conspecifics. Similar to the congener *C. scaber*, *C. ales* exhibits an off-response (shadow reflex) and an on-response (light reflex). In field observations, a shadow caused a significant increase in flash rate from a mean of 3.9 Hz to 4.7 Hz (*P*=0.0016). In laboratory trials, a looming stimulus, which increased light intensity, caused a significant increase in flash rate from a median of 1.8 Hz to 2.2 Hz (*P*=0.0001). Morphological analysis of the eyes of *C. ales* revealed coarsely-packed photoreceptors lacking sophisticated structure, resulting in visual resolution that is likely too low to detect the flashing of conspecifics. As the eyes of *C. ales* are incapable of perceiving conspecific flashing, it is likely that their vision is instead used to detect predators.

## Abstract (precis)

**Summary:** ‘Disco’ clams are known for their vivid flashing displays. Their vision was examined to see if their display is visible to other disco clams as a signal.

## INTRODUCTION

Marine invertebrates communicate through diverse channels, including visual (Adams and Caldwell, 1990; Adams and Mesterton-Gibbons, 1995; Marshall et al., 2015), chemical (Daloze et al., 2013; O'Connell, 1986; Scheuer, 1977), tactile (Caldwell and Dingle, 1976; Knowlton, 1996), and acoustic (Caldwell, 1979) signals. Visual communication can be achieved through the use of light (including UV, fluorescence, and polarization) (Bok et al., 2014; Chiou et al., 2008; Cronin, 2006; Miya et al., 2010), color (Kohda et al., 2005; Sugimoto, 2002), countershading (Penacchio et al., 2014), and shape (Allen et al., 2014; Baker, 2010). The optical receptors needed to detect visual signals exhibit great diversity in size, shape, number and complexity (Land and Nilsson, 2012). The rapid diversification of photoreceptors in invertebrate taxa was driven by the emergence of visually guided, fast locomotion during the Cambrian explosion (approximately 530-520 million years ago) (Nilsson, 1996). When considering the evolution of vision, simple light-sensitive cells can evolve to a complex camera-type eye in a mere few hundred thousand years (Nilsson and Pelger, 1994), which may explain the extreme diversity of vision throughout the animal kingdom.

Mollusks (bivalves, gastropods, cephalopods, chitons, etc.) have the most diverse eye morphologies of any phylum (Serb and Eernisse, 2008). Several eye types have evolved within Mollusca: pit eyes can differentiate between light and shade but do not form images, and exist in some bivalves and gastropods; pinhole eyes, which provide the directionality of light but poor image quality, exist in the nautilus and the giant clam; compound eyes, which have poor image resolution but detect motion, exist in some ark clams; mirror eyes, which are image-forming, exist in scallops; camera-type eyes, the most complex eye type which forms detailed images, exist in cephalopods (Land and Nilsson, 2012). Eye placement varies in mollusks, including cephalic (head) eyes, mantle eyes, and eyes embedded in shells (Serb and Eernisse, 2008). The range of eye size within Mollusca is vast: from <100 µm in chitons (Speiser et al., 2011) to 25-40 cm in giant squid (Roper and Boss, 1982).

In bivalves, eye type varies between taxa, including photoreceptive cells in the mantle, pit eyes, mirror eyes, and compound eyes. Some bivalves possess multiple types of eyes along the mantle, such as ark clams (family Arcidae), which have both pit eyes and compound eyes (Nilsson, 1994; Patten, 1887; Waller, 1980). The number of eyes in bivalves also varies widely. In the family Cardiidae, some species have eye numbers in the tens (Morton, 2008), while others (e.g. giant clams) have eye numbers in the thousands (Wilkens, 1986). The only bivalve eyes that are known to form images are the mirror eyes of scallops (Land, 1965) and swimming scallops possess better vision than sessile scallops (Speiser and Johnsen, 2008a). Scallop eyes have multi-layer reflectors made of guanine crystals which redirect light to the double-retina (Land, 1966). Scallops can detect moving objects (Buddenbrock Von and Moller-Rache, 1953), direct swimming accordingly (Hamilton and Koch, 1996), and adjust the opening of their valves based on the size and speed of particles in the water (Speiser and Johnsen, 2008b).

Bivalves are relatively immobile, so it is thought that their eyes are used primarily to detect predators and trigger a defensive response (Nilsson, 1994). The shadow reflex, which is a neural and physiological reaction to a sudden decrease in light intensity, is widespread in both freshwater and marine bivalves (Morton, 2008). Most bivalve eyes are located on the mantle (pallial eyes), and are found on (i) the outer mantle fold (Arcoidea, Limopsoidea, Pterioidea, and Anomioidea), (ii) the middle mantle fold (Pectinoidea and Limoidea), and (iii) the inner mantle fold (Cardioidea, Tridacnoidea, and Laternulidae). Generally, pallial eyes measure the amount and direction of light, giving a distribution of light in the immediate environment of the bivalve. However, this does not mean they can perceive an image (Morton, 2008), with the exception of the mirror eyes of scallops (Land, 1965).

*Ctenoides ales* (Finlay, 1927) are sessile Indo-Pacific bivalves (Limidae) that are found attached inside small crevices on coral reefs using byssal threads. They are found from 3 to ≥50 m, where light intensity decreases with depth (Dougherty, 2016). They are the only bivalves known to exhibit a flashing display. The display is so vivid that it has been confused for bioluminescence (Mikkelsen and Bieler, 2003; Okubo et al., 1997), but is actually the result of structural reflection (Okutani, 1994) from silica nanospheres in the mantle edge (Dougherty et al., 2014). No studies of *C. ales* have elucidated the visual acuity or morphology of their eyes, or whether they are capable of resolving flashing in conspecifics. Here, the eyes of *C. ales* were studied to help understand the role of vision in their life history, and to determine whether the flashing display is visible to other *C. ales* organisms as a signal.

Conspecific recruitment was considered as a potential function of the flashing display of *C. ales*, as 60% of organisms were found in groups (930 cm^2^±10 cm) of 2-4 individuals (*n*=106) during field sightings. Size differentials among the organisms (1-8 cm shell height) suggested settlement was asynchronous (Dougherty et al., 2014). Chemical cues are used in settlement of larval bivalves (García-Lavandeira et al., 2005; Green et al., 2013; Harvey et al., 1997; Mesías-Gansbiller et al., 2013; Wassnig and Southgate, 2012), but many species possess light-sensitive eyes during their pediveliger stage, which precedes settlement (Carriker, 1990). Therefore, vision was considered as an alternative or additional settlement signal for *C. ales* bivalves.

The eyes and dermal photosensitivity of the congener *Ctenoides scaber* (Born, 1778) have been well studied (Bell and Mpitsos, 1968; Dakin, 1928; del Pilar Gomez and Nasi, 1995; Gomez and Nasi, 1994; Mpitsos, 1973; Nasi, 1991; Wilkens, 2008). Dermal photoreceptors located near the eyes of *C. scaber* generate only off-responses (to shadows). The eyes have both off- and on-responses. The off-response (primary inhibition) occurs in hyperpolarized, ciliary cells, and the on-response occurs in depolarized rhabdomeric cells (Mpitosos, 1973). There are no synaptic interactions between the two, as the proximal and distal retinas are functionally independent of one another (Mpitosos, 1973).

Morton (2000) showed that *Ctenoides mitis* (Lamarck, 1807, previously *C. floridanus*) possess ∼18 eyes at the base of the pallial tentacles with a lens, collagen overlap (which seals the lens), cornea, transverse fibers (which connect the middle mantle fold to the epithelia of the inner surface of the transparent haemocoel), pigmented cells and vacuolated cells (which make up the base of the retina), and an optic nerve. The eyes largely resemble those of other bivalves in the super-families Arcoida and Limopsoidea (Waller, 1980). The only other research on limid bivalves eyes include descriptions of the eye morphology of *Acesta excavata* (Fabricius, 1779) (formerly *Lima excavata*) (Schreiner, 1896) and *L. vulgaris* (Link, 1807, formerly *L. squamosa*) (Hesse, 1896), with updated studies on the latter by von Salvini-Plawen and Mayr (1977). These studies provided valuable information on eye morphology and physiology, but do not provide information to determine whether the eyes of *C. ales* are capable of perceiving the flashing of conspecifics.

The eyes of *Ctenoides* are a critical evolutionary link in increasing eye sophistication in bivalves. The eyes of *Ctenoides* are located on the middle mantle fold – bridging bivalves with pallial eyes on the outer mantle fold (beneath the shell), and those with the pallial eyes on the inner folds. The trend from outer fold to inner fold reflects what may represent increasing eye sophistication as the bivalve lineages become more derived (Morton, 2000). This theory is being tested in future studies involving a more well-sampled bivalve phylogeny and ancestral state reconstruction techniques by the authors. The goals of this study were therefore threefold: (i) to contribute to the knowledge of eye morphology in *Ctenoides*, and to determine whether there is variation within the genus in an evolutionary context; (ii) to determine whether the eyes of *C. ales* are capable of perceiving the flashing in conspecifics, a trait that is unique to the *C. ales* species; (iii) if *C. ales* is not capable of perceiving the flashing in conspecifics, to determine whether *C. ales* eyes may serve another purpose, such as detecting predators. To determine if eyes are used in detecting predators, shadow responses were examined *in situ* to determine if *C. ales* responded to decreases in light intensity by changing their flash rate, and looming trials were conducted in the laboratory to determine if *C. ales* responded to changes in light intensity (when a shadow was not present) by changing their flash rate.

## RESULTS

### Eye morphology

**Fig. 1.**
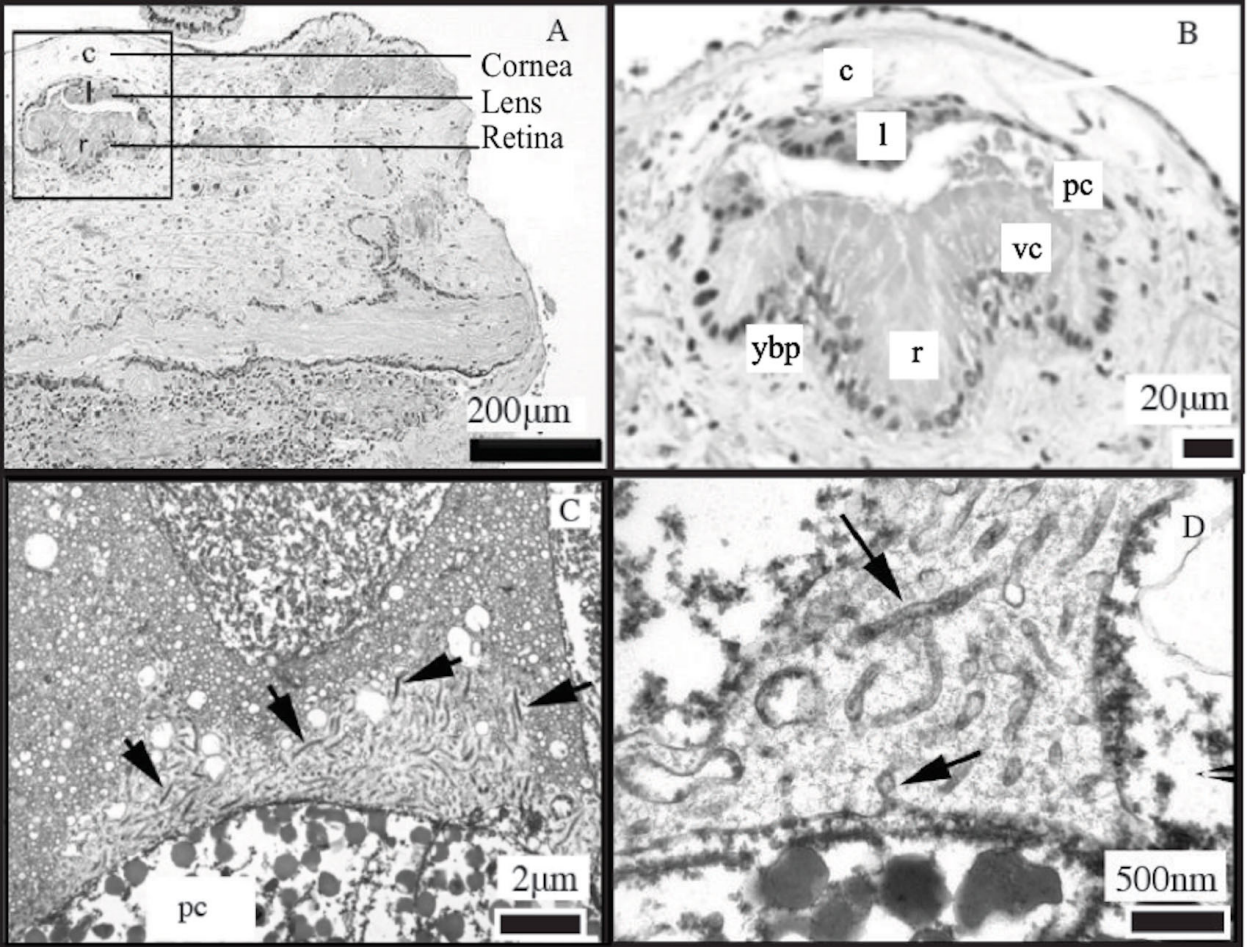
**Microscopy of the eyes of *Ctenoides ales*.** (A) Light microscopic photomicrograph showing the eye (box) of *C. ales* embedded in the tissues of the mantle. The cornea (c), lens (l), and retina (r) are labeled. Scale bar: 200 µm. (B) Light microscopic photomicrograph showing the cornea (c), lens (l), retina (r), pigment cells (pc), vacuolated cells (vc) and yellow brown pigment (ybp). Scale bar: 20 µm. (C) Transmission electron micrograph showing the photoreceptive cells (pc) and the rhabdomeric microvilli covering the inner surface of the cell (arrows). Scale bar: 2 µm. (D) Transmission electron micrograph at higher magnification than C showing the rhabdomeric microvilli (arrows) that are tangled and widely spaced. Scale bar: 500 nm.

The eyes of *C. ales* contained a lens, a clear cornea, and a retina (Fig. 1A). Pigmented cells and vacuolated cells, which together make up the retina, can be seen in Fig. 1B. The photoreceptive cells were positioned so that the rhabdomeric microvilli pointed in the direction of the cornea (Fig. 1C). The rhabdomeric microvilli averaged 2.82 μm height by 0.08 μm width (±0.34 μm, 0.01 μm) and were spaced an average of 0.13 μm apart (±0.03 μm). They appear tangled and are widely spaced (Fig. 1D), with no complex structural packing. The small size of the eye combined with the irregular shape of the retina makes it difficult to determine an accurate focal length or receptor separation length, which would allow for a calculation of the inter-receptor angle and maximum resolvable spatial frequency (Land and Nilsson, 2002). The morphological description of the eye of *C. scaber* by Bell and Mpitsos (1968) and of *C. mitis* by Morton (2000) are similar to the morphology of the eye of *C. ales* found in this study.

### Shadow reflex

**Fig. 2.**
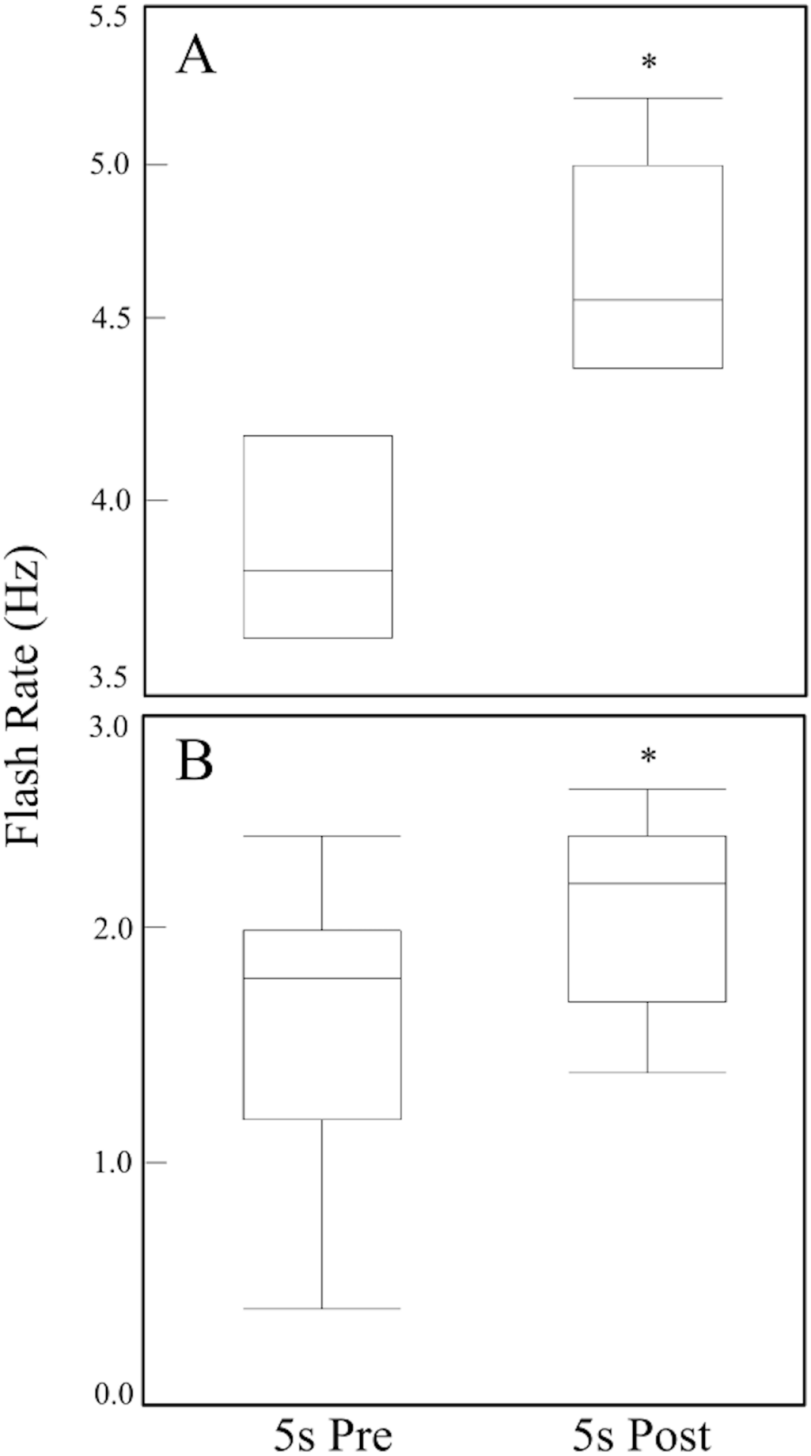
**Changes in flash rate (Hz) of *Ctenoides ales* in response to shadow and looming stimuli.** (A) Measured for 5 s before and 5 s after a shadow obscured *C. ales in situ* (*n*=7). (B) Measured for 5 s before and 5 s after looming stimulus (25 cm^2^ Styrofoam) in laboratory (*n*=18). Flash rates in both experiments increased significantly after stimulus. Box and whiskers indicate minimum, maximum, median, and upper and lower quartiles; asterisk indicates a significant increase; Mann-Whitney tests were used; (A) *P*=0.0016, (B) *P*=0.0001.

When a shadow obscured *C. ales in situ*, their flash rate (Hz) increased significantly in the 5 s following the stimulus (Mann–Whitney, *P*=0.0016, *n*=7). The flash rate increased from a pre-stimulus median of 3.8 Hz to a post-stimulus median of 4.6 Hz (Fig. 2A). The sources of the shadows in the video are unknown, as video was taken without scuba divers present, and the animals causing the shadows were not recorded by the camera.

### Looming trials

When a looming stimulus (a white, 25 cm^2^ rectangle) was moved toward *C. ales*, their flash rate (Hz) increased significantly in the 5 s following the stimulus (Mann–Whitney, *P*=0.0001, *n*=18). The flash rate increased from a pre-stimulus median of 1.8 Hz to a post-stimulus median of 2.2 Hz (Fig. 2B).

## DISCUSSION

Morphological analysis of the eyes of *C. ales* revealed a lack of complex structure and coarsely-packed photoreceptors. Without refined structure to detect the direction of light (Land and Nilsson, 2012), it is very unlikely that *C. ales* is capable of resolving the flashing of other *C. ales* organisms, as the flashing display is small (mantle reflective strip <1 mm wide, <3 cm long) and rapid (≤5.2 Hz), and their visual resolution is likely very low. These results suggest the flashing of *C. ales* is not a signal intended for conspecifics, and that the clustered groupings observed in the field may be the result of a chemical cue that aids in juvenile settlement, similar to other bivalves (García-Lavandeira et al., 2005; Green et al., 2013; Harvey et al., 1997; Mesías-Gansbiller et al., 2013; Wassnig and Southgate, 2012). *C. ales* responded to both a looming stimulus (increased light intensity) and shadows (decreased light intensity) by significantly increasing their flash rate. The morphological similarity of the eye of *C. ales* to the eye of the congener *C. scaber* suggests that *C. ales* eyes are also responsive to light increases and decreases (Mpitosos, 1973). It is therefore probable that their vision is used for predator detection, as in *C. scaber*.

The eyes in the family Limidae, to which *C. ales* belongs, have not been investigated as thoroughly as other families such as Pectinidae (scallops), likely because they are much smaller, fewer, and more hidden within the mantle tentacles (Morton, 2008). Of the ten genera in Limidae, only *Limaria* do not possess eyes (Morton, 1979). Whether this is related to their unique defensive mechanism of tentacle autotomy and their inability to fully retract their tentacles to close their shell (Morton, 1979), is unknown. Of the remaining genera, only some species possess pallial eyes (Dakin, 1928), including *Lima* and *Ctenoides*. The morphological structure of the eyes of *C. ales* is remarkably similar to the structure of the eyes of *C. mitis* (previously *C. floridanus*) (Morton, 2000). The similarity is important for two reasons. First, it suggests that although *C. ales* evolved a unique flashing display that no other bivalve exhibits, there does not seem to be any corresponding change in eye morphology. Second, there is a drastic difference in eye complexity between these *Ctenoides* species and *Lima vulgaris* (formerly *Lima squamosa*) (von Salvini-Plawen and Mayr, 1977) from a closely related genus. The eyes of *L. vulgaris* are described as everse pinhole eyes, with little more than a vitreous mass and retinal cells. The difference in eye complexity between these two genera is notable, and future studies would benefit from phylogenetic comparative analyses of eye evolution within Limidae.

Due to the variety of eye types in Limidae, eye evolution within this group needs to be solved using a more comprehensive phylogeny and detailed eye studies of more limid bivalves. Many molecular studies elucidate the phylogenetic relationships of a few limid bivalves (González et al., 2015; Giribet and Wheeler, 2002; Plazzi et al., 2011; Serb, 2008), but none include a comprehensive analysis of the relationships among *Ctenoides*, *Lima*, *Limaria*, and the outgroup *Pectinidae* (scallops). This line of research should be of great interest in the context of invertebrate eye evolution and convergent evolution of complex morphological traits, and is being pursued in future studies by the authors.

Since the flashing display of *C. ales* is not visible to conspecifics, other purposes for the flashing must be considered. Elaborate displays in nature are generally used for three purposes: (i) to attract conspecifics (Beekman et al., 2016; Lange et al., 2013; O'Day, 1974), which was considered in this study; (ii) to lure prey items (light, color, or mimicry designed to attract food items) (Hanlon and Messenger, 1996; Johnsen et al., 1999; O'Day, 1974; Shallenberger and Madden, 1973; Shimazaki and Nakaya, 2004), which was tested in a separate study (Dougherty et al., 2016); or (iii) to communicate aposematism (warning coloration signaling distastefulness) (Hanlon and Messenger, 1996; Williams et al., 2011). The significant increase in the flash rate of *C. ales* when an increase or decrease in light occurs, combined with the visibility of the flashing of *C. ales* to many of its potential predators (Dougherty et al., 2014), suggest the flashing may function as a warning (aposematic) signal. This hypothesis is being considered in future studies.

## MATERIALS AND METHODS

*C. ales* bivalves were obtained from Blue Zoo Aquatics (Hawthorne, CA, USA). All bivalves had a shell height of >4 cm. The size at which this species changes from male to female (protandrous hermaphroditism) is unknown. Prior to experiments, the bivalves were housed in a single 375-liter tank with a water temperature of 25-27°C. They were given a 12 h light:12 h dark light regime, and each bivalve was given 1 ml of phytoplankton mixture (Phytofeast©, Campbell, CA, USA) three times per week. Experiments were conducted during the day, as the mantle of *C. ales* (which causes the flashing) is reflective, and therefore only visible when there is ambient light.

### Eye morphology

To examine the structure of the eyes of *C. ales*, two eyes (∼1 mm^2^) from one *C. ales* bivalve were removed and fixed in 2.5% glutaraldehyde with a sterile seawater buffer. One of the eyes was embedded in paraffin, sectioned at 5 µm and stained with hematoxylin, which stains nucleic acids, and eosin, which stains protein. The other eye was treated with 1% OsO_4_ in PBS for 2 h at room temperature followed by three 10-min washes with a 0.1 M sodium acetate buffer. Tissues were then stained with 2% uranyl acetate in a sodium acetate buffer for 1 h at room temperature, washed in buffer, dehydrated in a graded ethanol series (40–100%), and infiltrated with propylene oxide-812 resin (1005 Embed 812; EMS, Fort Washington, PA, USA). The sample was embedded with fresh 100% 812 resin in molds and polymerized in a 60°C oven for 36 h. Ultrathin sections (90 nm) were analyzed using a JEOL 100CX electron microscope (JEOL Ltd., Tokyo, Japan).

### Shadow reflex

To determine whether *C. ales* responded to shadows, video was taken of *C. ales* individuals *in situ* in Indonesia (Lembeh Straight, 1°27′ N, 125° 14′E and Kri Island, 0° 34′S, 130° 40′E). Go Pro Hero3^®^ video cameras were attached to 4 kg weights using zip ties, and an underwater light (Light & Motion Gobe^®^, Marina, CA, USA) was aimed at the area of interest in which the *C. ales* individual was attached. The depth where recordings were taken varied from 10-15 m. A total of 16 h and 57 min were recorded without scuba divers present. The camera setups were left in place and then retrieved on subsequent dives. A total of seven events where a shadow passed across the clam were recorded. Video was analyzed 5 s before and 5 s after the shadow, and the flash rate (Hz) was recorded.

### Looming trials

**Fig. 3.**
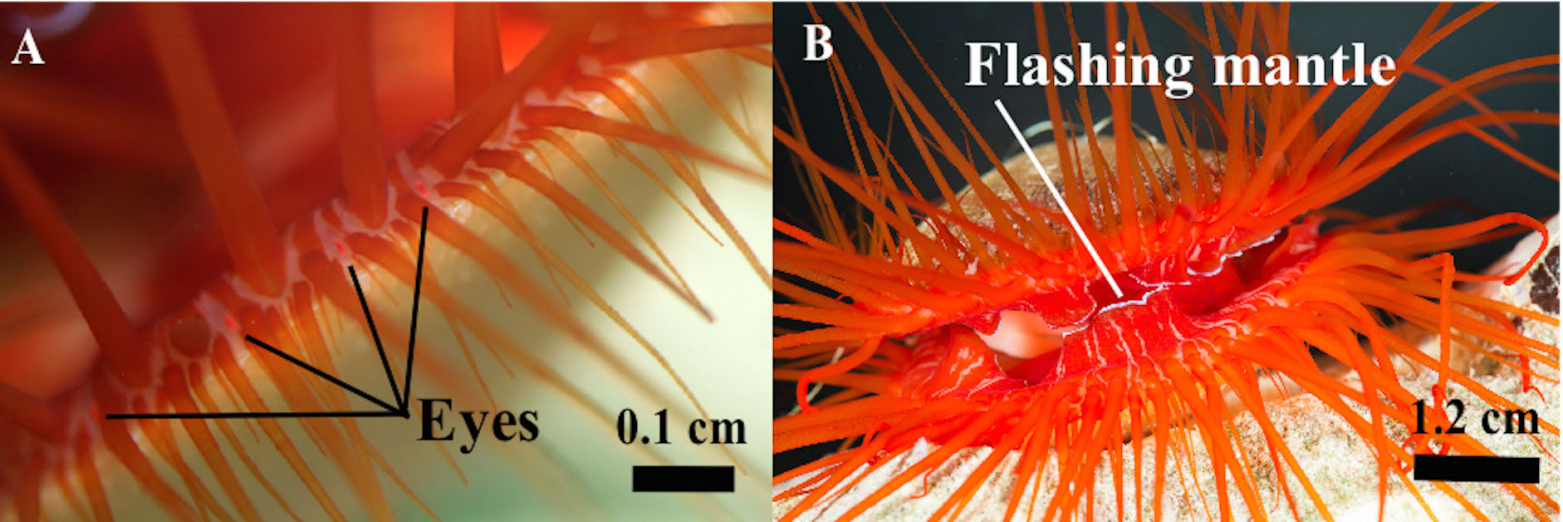
**The eyes of *Ctenoides ales*.** (A) Macro photograph of the eyes of *C. ales*. Scale bar: 0.1 cm. Photo credit: L. Dougherty. (B) Whole-organism photograph of *C. ales*, showing the location of the flashing line on the mantle. Scale bar: 1.2 cm. Photo credit: R. Caldwell.

To test whether the eyes of *C. ales* (Fig. 3A) were capable of detecting movement of an object (such as a predator) when a shadow was not present, a looming stimulus (Cartron et al., 2013a,b; Gallagher and Northmore, 2006; Herberholz and Marquart, 2012) was used. The behavioral reaction measured in this study was the flash rate (Hz) of *C. ales* (Fig. 3B). Looming trials were conducted in a 37-liter tank with black boards surrounding all sides to block external stimuli, except the front, where the stimulus was presented and the flash rate of *C. ales* was recorded on video (Sony Cybershot^®^ DSC-W7 Digital Camera). A white, 25 cm^2^ rectangle was used as the stimulus. The stimulus was quickly (≤1 s/30 cm) moved toward the experimental clam (*n*=18) 30 s after video began recording. The stimulus stayed in place for 30 s and then recording was stopped. Preliminary trials indicated changes in flash rates were brief (≤5 s), so the flash rate was analyzed by viewing the recording 5 s before and 5 s after the looming stimulus, and recording the flash rate (Hz).
